# Deformable mirror-based axial scanning for two-photon mammalian brain imaging

**DOI:** 10.1117/1.NPh.8.1.015003

**Published:** 2021-01-01

**Authors:** Alba Peinado, Eduardo Bendek, Sae Yokoyama, Kira E. Poskanzer

**Affiliations:** a University of California, San Francisco, Department of Biochemistry and Biophysics, San Francisco, California, United States; b National Aeronautics and Space Administration, AMES Research Center, Moffet Field, California, United States; c Kavli Institute for Fundamental Neuroscience, San Francisco, California, United States

**Keywords:** two-photon microscopy, deformable mirror, axial scanning, brain imaging, astrocyte

## Abstract

**Significance:** To expand our understanding of the roles of astrocytes in neural circuits, there is a need to develop optical tools tailored specifically to capture their complex spatiotemporal ${\mathrm{Ca}}^{2+}$ dynamics. This interest is not limited to 2D, but to multiple depths.

**Aim:** The focus of our work was to design and evaluate the optical performance of an enhanced version of a two-photon (2P) microscope with the addition of a deformable mirror (DM)-based axial scanning system for live mammalian brain imaging.

**Approach:** We used a DM to manipulate the beam wavefront by applying different defocus terms to cause a controlled axial shift of the image plane. The optical design and performance were evaluated by an analysis of the optical model, followed by an experimental characterization of the implemented instrument.

**Results:** Key questions related to this instrument were addressed, including impact of the DM curvature change on vignetting, field of view size, image plane flatness, wavefront error, and point spread function. The instrument was used for imaging several neurobiological samples at different depths, including fixed brain slices and *in vivo* mouse cerebral cortex.

**Conclusions:** Our implemented instrument was capable of recording z-stacks of 53  μm in depth with a fine step size, parameters that make it useful for astrocyte biology research. Future work includes adaptive optics and intensity normalization.

## 1 Introduction

Two-photon (2P) microscopy^1^^,^^2^ is a widely used technique for imaging in high-scattering media, such as the mammalian brain. Because 2P microscopy uses near-infrared (NIR) light, which has low scattering and low absorption in biological tissues, it enables imaging at biologically significant penetration depths. Much current 2P microscopy takes advantage of genetically encoded calcium (${\mathrm{Ca}}^{2+}$) indicators to record neural activity in the brain.^3^ While most 2P ${\mathrm{Ca}}^{2+}$ imaging targets neuronal activity, other cell types also exhibit spontaneous ${\mathrm{Ca}}^{2+}$ dynamics. These include astrocytes, the largest class of non-neuronal brain cells which are largely electrically silent but which exhibit spatiotemporally rich ${\mathrm{Ca}}^{2+}$ dynamics.^4^^,^^5^ These astrocytic ${\mathrm{Ca}}^{2+}$ dynamics have been shown to be critical for astrocytic regulation of brain states related to sleep^4^^,^^6^ and attention.^7^ To expand our understanding of the roles of astrocytes in neural circuits, there is a need to develop optical tools that are tailored specifically to capture astrocytic ${\mathrm{Ca}}^{2+}$ physiology. These tools include image analysis algorithms to quantitatively evaluate the irregular and propagative ${\mathrm{Ca}}^{2+}$ activity exhibited by astrocytes, as well as instrumentation^8^ for recording these spatiotemporally ${\mathrm{Ca}}^{2+}$ dynamics.

2P microscopy is a laser scanning technique in which a xy scanner is used to move the beam focus across a sample, obtaining a 2D image. Recently, a variety of volumetric 2P systems that enable population-wide imaging at multiple z-planes have been reported to enable the study of neuronal activity across brain tissue depths.^9^ One group of these systems applies technologies that sequentially move the focus along the z axis and includes piezo-actuated objectives,^8^^,^^10^^,^^11^ electro-tunable lenses,^12^^–^^14^ and remote focusing.^15^^–^^17^ Because their settling times are on the order of a few milliseconds, these technologies are mostly limited to imaging ${\mathrm{Ca}}^{2+}$ activity at a few defined z planes. Other systems use faster axial scanners such as an acoustic-optical scanner^18^ or an ultrasound-driven lens,^19^ but they can suffer from focusing aberrations. Another solution to imaging in depth is the technique of implanting microprisms in the brain to image orthogonal to the skull surface while still using a traditional xy scanner.^20^^,^^21^ For non-neuronal cell types, including astrocytes, which become reactive upon injury, this type of invasive technique may only allow the study of pathological—rather than physiological—dynamics. Still other techniques for 2P imaging beyond two dimensions are based on spatially multiplexing the beam focus at different depths to carry out volumetric 2P *in vivo* brain imaging. Some use a spatial light modulator (SLM) to split one beam into several beamlets at different depths,^22^ while others use Bessel beams^23^^,^^24^ to simultaneously excite an axially elongated focus. However, the recorded signal for these instruments is a 2D projection of a 3D volume. Therefore, they depend on source-separation algorithms^25^ to retrieve individual signal contributions and built expressly for neuronal somatic activity. However, astrocytic ${\mathrm{Ca}}^{2+}$ activity exhibits differential characteristics from neuronal ${\mathrm{Ca}}^{2+}$ activity with more complex and variable spatial dynamics, and these characteristics render current source-separation algorithms unusable for astrocytes. For all these reasons, new imaging techniques are necessary to record activity of neuronal and non-neuronal cell types across layers with a high z-resolution.

Here, we use a deformable mirror (DM) as an active optical element to achieve axial scanning for *in vivo* brain imaging. A DM has a surface that can be dynamically shaped in order to control the light wavefront and microelectromechanical systems (MEMS)-based DMs have been shown to be capable of changing their surface shape at KHz rates.^26^ Thus, using this technology in a 2P microscope has the potential for imaging multiple planes at different depths without compromising the volumetric acquisition rate. In this paper, we implement a MEMS DM-based axial scanning for 2P microscopy to study any ${\mathrm{Ca}}^{2+}$-signaling brain cell, including astrocytes and neurons, complementing the contributions of others in the field.^27^^–^^29^ Our goal was to evaluate the optical design and performance of the DM scanning system for axial 2P brain imaging. To do so, we analyzed the optical model and experimentally characterized the implemented instrument. Analyses performed include vignetting characterization, field-of-view (FOV) size dependence with DM curvature, characterization of the point spread function (PSF), wavefront error (WFE) mapping, and axial scanning range capability.

The outline of this paper is as follows. In Sec. 2, we introduce the concept of using the DM as an active optics element for axial scanning, present a model, and experimentally calibrate the scanning module performance. In Sec. 3, the optical setup of a 2P microscope with the DM-based axial scanning is described, its optical model is analyzed, and its implementation is experimentally characterized. In Sec. 4, experimental measurements obtained with the instrument are shown, including imaging of pollen grains, fixed brain slices, and *in vivo* mouse cerebral cortex. Finally, Sec. 5 summarizes the main results of this work.

## 2 Deformable Mirror-Based Axial Scanning

### 2.1 Concept

A DM is a reflective device that can modify the phase of an incident electromagnetic wave by changing the shape of the mirror surface. The DM is composed of a two-dimensional array of actuators in which their axial position (z) is adjusted individually to generate a desired shape. The intrinsic reflective operation of the device results in a wavefront change twice as large as the surface stroke applied. There are segmented DMs (composed of independent flat mirror segments) and continuous DMs (with actuators fixed to the back side of a continuous reflective membrane). Nowadays, many technologies use DMs as the key element for applying active and adaptive optics in diverse applications, such as astronomy,^30^ ophthalmology,^31^ and biology.^32^^,^^33^ In applications requiring smooth wavefront control, such as in our 2P microscope, a continuous DM is advised.

**Fig. 1:**
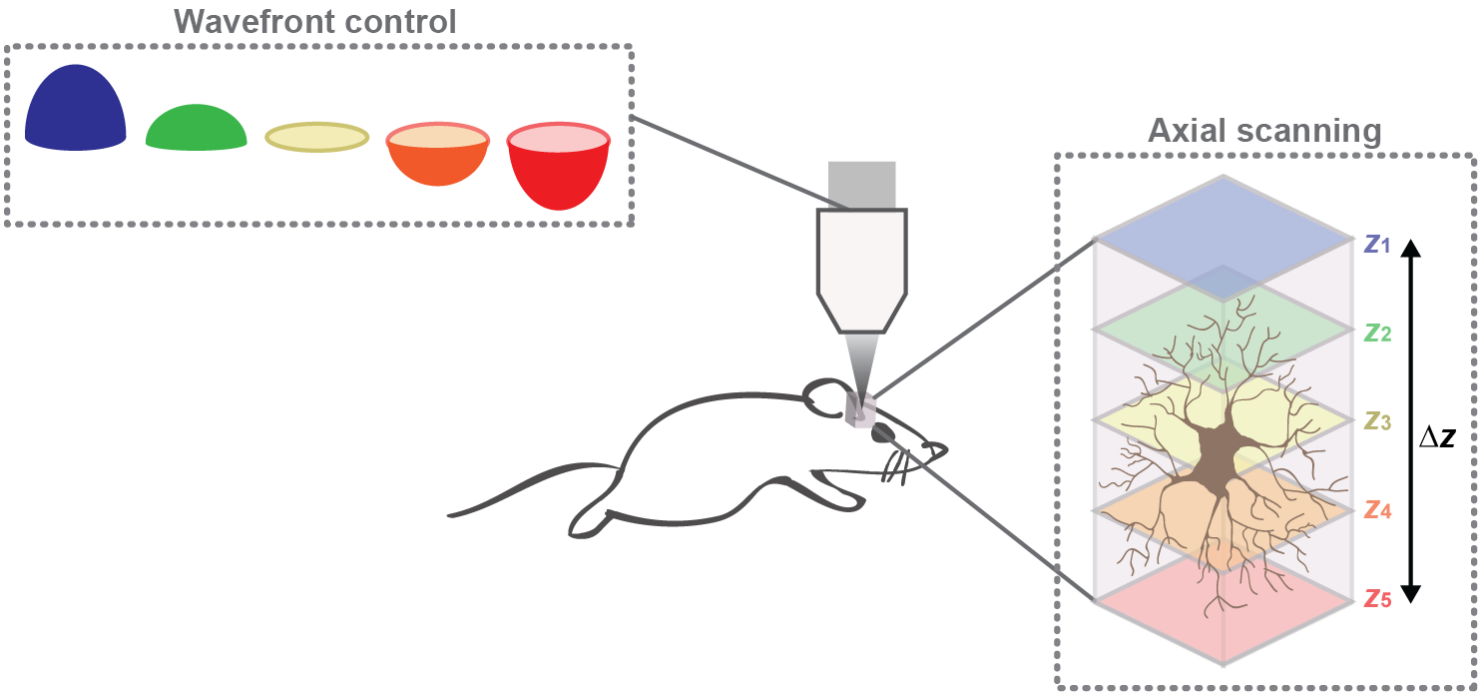
A DM is used for manipulating the light beam wavefront reaching the microscope objective entrance pupil and consequently causing different axial displacements of the beam focus over the specimen. This cartoon shows an example of an astrocyte imaged at five different depths.

In this work, we integrated a DM into a 2P microscope for recording brain activity at different depths. The axial scanning technique relies on using a DM as an active optics element to modify the light beam wavefront reaching the microscope objective entrance pupil. This generates an axial displacement of the microscope effective focal plane and consequently, it results in 2P imaging at different depths. The DM-based axial scanning concept for 2P brain imaging is schematized in Fig. 1.

### 2.2 Model

A lens modifies the phase of an incoming wavefront when light is transmitted through it. When the incoming beam is collimated, the lens will change the wavefront phase in such a way that at its exit pupil, there will be a spherical wavefront converging to a point a distance R, where R is the focal length of this lens. When a defocus term,

$$W(x,y)=-A(x^{2}+y^{2}),$$ (1)

is added to the original spherical wavefront of radius of curvature R, a focal shift will result given by^34^

$$\varepsilon_{z}=2R^{2}A.$$ (2)

where A is a parameter that defines the sign and amplitude of the defocus, and consequently it affects the axial focal shift. For the sign convention chosen in Eq. (1), when the optical path difference^34^ at the entrance pupil increases because of the addition of a positive defocus term (A<0), the spherical beam converges to a focal point moved toward the lens in the negative z axis direction. In contrast, for a negative defocus term (A>0), the focal point is moved away from the lens in the positive z axis direction.

The DM is placed at a conjugate plane of the microscope objective entrance pupil. In that way, when a quadratic surface is implemented on the DM, the reflected wavefront has a defocus term, which is reimaged at the entrance pupil plane of the microscope objective causing a controlled axial shift of the focal point.

We implemented a set of defocus distributions using Eq. (1), and we changed parameter A from −1 to 1, corresponding to mirror shapes ranging from a concave mirror to a convex mirror. The target surfaces were modeled with respect to a flat surface in which all actuators were at half of their maximum displacement (stroke). This flat surface was achieved by sending a given voltage map, known as flat correction, to compensate the intrinsic bow of the DM.

**Fig. 2:**
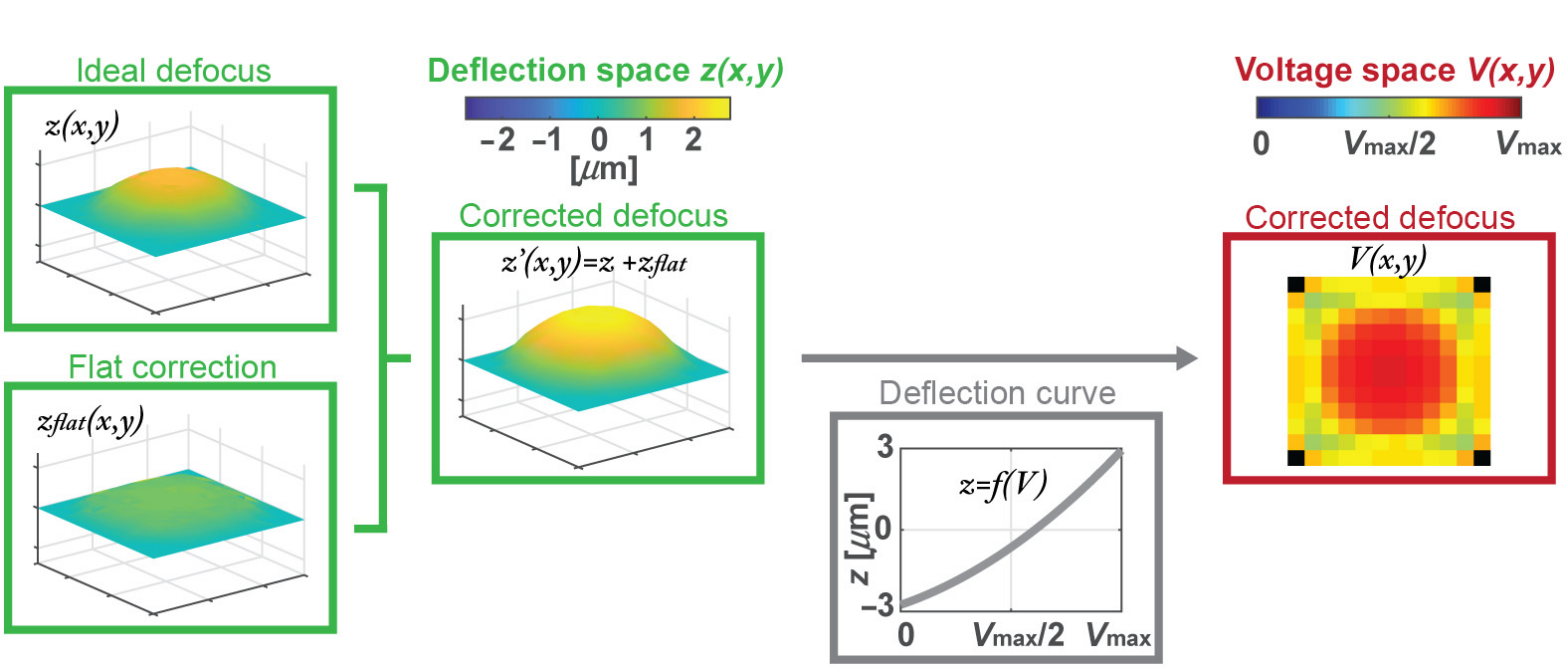
Flowchart describing the process from the ideal defocus deflection distribution to the voltage map sent to the DM, in which we consider the flat correction needed to flatten the intrinsic DM bow and the DM deflection curve.

In summary, the process for obtaining the final voltage map to be sent to the DM is the following (Fig. 2): First, a set of ideal defocuses, z(x,y), are modeled using Eq. (1) with a quadratic phase and a parameter A which defines the sign and magnitude of the radius of curvature. These functions, defined in the deflection space, are added to the flat map, $z_{\text{flat}}(x,y)$, and as a result, the corrected defocus deflection distribution, $z^{\prime }(x,y)$, is obtained. Then, these deflection distributions are converted from the deflection space to the voltage space using the deflection curve, a DM-specific function which relates voltage applied to a DM actuator with the actual deflection. Finally, we obtained the corrected defocus distribution in voltage units, also referred as voltage map.

The radius of curvature (R) of the DM defocus shape can be expressed as a function of the stroke (s) and the DM semi-aperture (x), using the sag equation of a spherical surface:^35^

$$R=\frac{x^{2}}{2s}+\frac{s}{2},$$ (3)

where the stroke is the peak-to-valley (PV) of the ideal defocus deflection distribution.

A variety of DMs are commercially available, and these vary by size, continuous versus segmented type, actuator pitch and stroke, number of actuators, and response time, with a trade-off between stroke and response time. For this project, we selected a continuous DM (Multi-5.5 DM, Boston Micromachines Corporation) of 12×12 actuators matrix with a maximum stroke of 5.5  μm and a total aperture of 4.95 mm, which offers a compromise between stroke and response time, which for this DM is smaller than 100  μs. The four actuators at the DM corners are fixed, for a total of 140 operative actuators. In addition, we used a circular mask of radius 5.5 actuators in which all actuators inside that mask are defined as the values given by protocol from Fig. 2, whereas actuators outside of it are assigned to the flat map value. This mask helps to ensure the correct implementation of the desired shape inside the pupil even though the constraint of the bordering actuators which are fixed. Theoretically, our DM can implement from a concave mirror of radius of curvature −746  mm (A=−1), to a convex mirror of +746  mm of radius of curvature (A=+1).

### 2.3 Experimental Characterization

#### 2.3.1 Free propagation

**Fig. 3:**
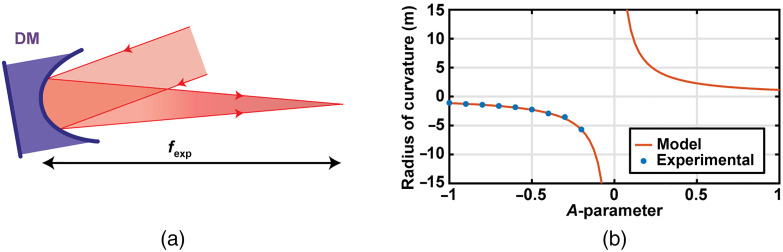
(a) A collimated light beam is focused at a distance $f_{\text{exp}}$ by the DM with a concave shape. (b) Experimental and theoretical DM radius of curvature as a function of the A-parameter.

In this section, we describe an experimental characterization of the focusing capability of the DM when implementing a concave mirror (R<0) with the DM. To this aim, we implemented the following setup [see Fig. 3(a)]. A collimated beam (measured by a Shack–Hartmann sensor) illuminates the DM, and the reflected beam is freely propagated. Then, we measured the distance in which the beam was focused from the DM. In other words, we measured the experimental focal length, $f_{\text{exp}}$, of the DM acting as a converging lens. From the focal length measurements, we calculated the radius of curvature:

$$R=2f_{\exp}.$$ (4)

The DM radius of curvature as a function of the A-parameter is plotted in Fig. 3(b). Blue points correspond to the experimental values using Eq. (4). In addition, the continuous orange line corresponds to the modeled data using Eq. (3). When A<0, radius of curvature is negative, corresponding to a concave mirror. Here, the reflected beam converged at a given distance and $f_{\text{exp}}$ was measured. In contrast, when A>0, radius of curvature is positive, corresponding to a convex mirror, and the light beam diverged after the DM reflection, creating a virtual focal point that cannot be directly measured. Finally, when A=0, corresponding to a flat DM, the radius of curvature is infinite. Thus, the DM did not affect the convergence of the reflected beam. We observe a good agreement between experimental and model data, proving that the DM manipulates the wavefront as expected when using concave-like curvatures (A<0). This experiment was conducted illuminating with a red diode laser at 635 nm (CPS635R, Thorlabs) but because the DM is achromatic due to its reflective operation, these results are valid for other wavelengths.

#### 2.3.2 Wavefront calibration

After we validated the focusing DM capability for concave mirror shapes, we tested arbitrary DM defocus shapes by measuring the actual reflected beam wavefront with a Shack–Hartmann wavefront sensor.^36^ Any wavefront can be defined as a linear combination of the Zernike polynomials,^34^ a basis of optical aberrations whose terms are orthogonal over the interior of a unit circle.

**Fig. 4:**
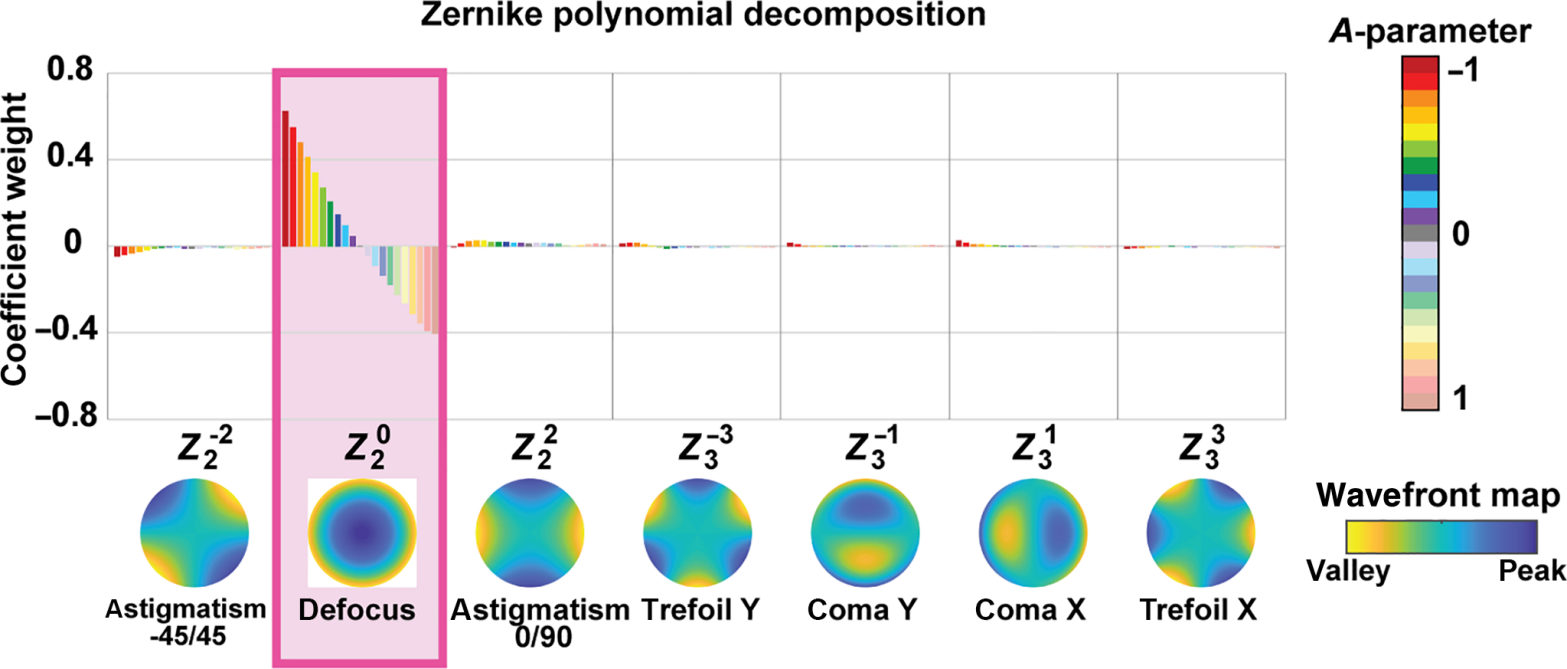
Zernike polynomial decomposition for 21 different DM shapes (A-parameter) measured with a Shack–Hartmann wavefront sensor.

The experiment consisted of illuminating the DM with a collimated laser beam at 635 nm (CPS635R, Thorlabs), and immediately measuring the reflected beam wavefront using a Shack–Hartmann wavefront sensor (WFS30-5C, Thorlabs). Twenty-one different shapes were implemented with the DM, varying the A-parameter from −1 to 1 in steps of 0.1. The wavefront was measured as a function of the Zernike polynomial terms. The weights measured for the different Zernike coefficients are plotted in Fig. 4 for the 21 DM shapes. Figure 4 also includes in the horizontal axis the wavefront map representation of each Zernike term in a unitary circle. The defocus term, highlighted with a pink box in Fig. 4, is linearly dependent with the A-parameter, whereas the other Zernike terms are negligible. Measured defocus Zernike coefficients are consistent with the values predicted by the model,^35^ with a 13% standard deviation error and a maximum deviation of 22% at the end of the range (corresponding to A=+1 and −1). There are several contributors to the non-linearity associated to this error, such as interactuator coupling and mirror-edge effects.

## 3 Two-Photon Microscope with Deformable Mirror-Based Axial Scanning

### 3.1 Optical Setup

Our 2P microscope is a custom-built instrument which combines a traditional upright fluorescence microscope (BX51WI, Olympus) with three additional modules to enable 2P microscopy: the illumination module composed of the pulsed laser and the relay optics, the scanning module where the DM and scanning galvanometer mirrors (GMs) are housed, and a detection system that uses photomultiplier tubes (PMTs).

**Fig. 5:**
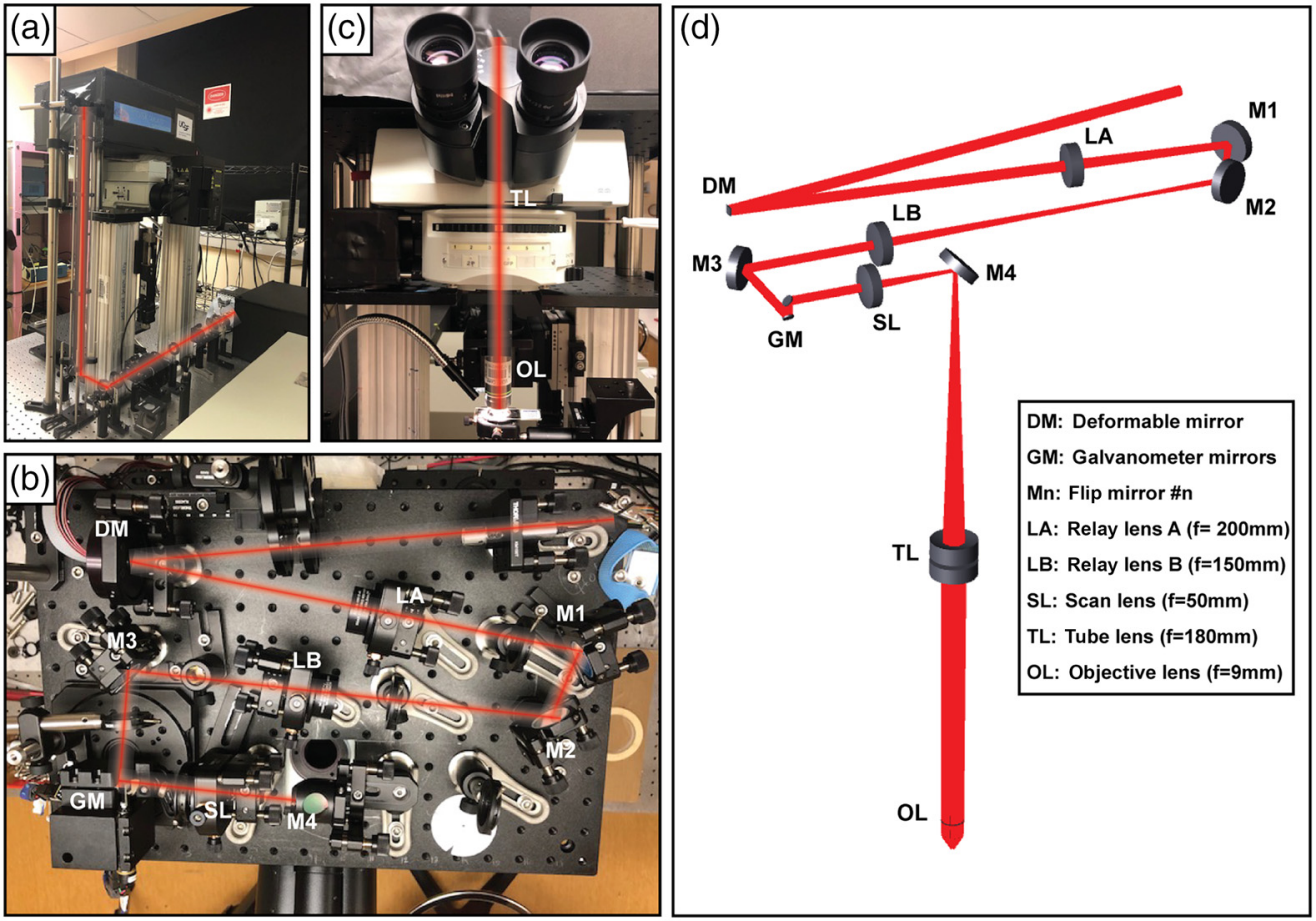
Pictures of the implemented 2P microscope. (a) Illumination module, including laser, Pockels cell, relay optics, and a periscope to bring the optical beam from the optical table where the laser is placed to the scanning module level. (b) Scanning module, including the DM and the GM, in addition to folding mirrors and lenses. (c) Front perspective of the microscope, in which the light beam is taken from the scanning module down through the tube lens and the objective lens to the sample. (d) Optical model of the 2P microscope illumination path.

First, Fig. 5(a) corresponds to a picture of the illumination module. As illumination source, we use a Ti:sapphire laser (Mai Tai HP, Spectra Physics) with a tunable wavelength from 690 to 1040 nm, which generates pulses of <100  fs with a pulse rate of 80 MHz. In addition, a Pockels cell (350–80, Conoptics) controls the beam intensity illuminating the sample. A beam relay system delivers the laser beam with the adequate magnification at a reimaged pupil located at the surface of the DM. Then, the scanning module [Fig. 5(b)] is composed of the DM and the GMs, which perform, respectively, the axial and lateral scanning over the sample. The DM is used to manipulate the light beam wavefront and consequently, to generate different displacements of the beam focus along the z axis beyond the microscope objective [Fig. 5(c)]. Then, xy-scanning GMs are used (6215H, Cambridge Technology) to scan the focal point over the plane xy on the biological sample. A set of lenses are used to reimage the DM surface to a pupil plane located between the two GMs. A last reimage of the pupil is formed using another set of lenses on the entrance pupil of the objective. All lenses (Thorlabs) are achromatic in the range of 650 to 1050 nm. The lenses’ focal lengths were selected to reimage the pupil at the correct plane with an adequate beam diameter. All mirrors used are achromatic in the NIR range, optimized for ultrashort pulsed lasers (10B20EAG.1, Newport). Figure 5(d) shows the geometry of the illumination path including folding mirrors and focal lengths. A dichroic mirror is placed at 45 deg above the microscope objective to transmit wavelengths longer than 660 nm, allowing NIR light to illuminate the sample, but reflecting 2P emitted light in the visible range from the sample to the detector module. The detector module is composed of, first, an infrared blocking filter (ET680sp-2p8, Chroma) followed by a single-band bandpass filter (Semrock) and finally, a PMT (R6357, Hamamatsu). Finally, our system is controlled using ScanImage,^37^ an open-source MATLAB-based software for laser scanning microscopy focused on neurobiology. The microscope has the flexibility to be operated with different microscope objectives, but experimental data shown in this paper was obtained using a 20× water-immersion objective with NA 1.0 (XLUMPlanFLN, Olympus).

The 2P microscope optical layout, including pupil relays and the scanning system, was modeled in Zemax. First, lenses LA, LB, and the scan lens, [Fig. 5(d)] were modeled using the file provided by the manufacturer. An optical model for the Olympus tube lens was found in the literature^38^ and incorporated in the optical model of the instrument. Additionally, folding mirrors were included in the design to fold the optical path in the space available. These folding mirrors and the GMs were modeled using real dimensions. Then, the DM was modeled as a mirror with an adjustable radius of curvature. Because Olympus does not provide the optical design of the microscope objective due to intellectual property protection, we modeled this element as a paraxial lens with the specified focal length and entrance pupil diameter.

### 3.2 Key Questions During Design and Implementation

During the design and implementation of this instrument, several questions arose concerning the optical design and performance of the 2P microscope when using DM-based axial scanning. Table 1 lists these questions, summarizes main outcomes, and lists the section in which the question is analyzed in detail. Questions are divided in two groups: those answered through the analysis of the optical model and those addressed with an experimental characterization of the implemented instrument. While some of these conclusions are implementation-dependent, they serve as guidelines for other 2P microscope implementations equipped with a DM for axial scanning.

**Table 1:** Optical design and performance questions about the 2P microscope with the DM-based axial scanning.

Key question	Outcome	See section
*Optical model analysis*		3.3
Is vignetting affected by a change in DM curvature?	For small FOVs (<∼600 μm for our implementation), no, but as the FOV increases, vignetting occurs partly due to the tube lens clipping caused by DM curvature-induced beam diameter changes.	3.3.1
Does the objective entrance pupil filling change when changing the DM curvature?	No, the beam diameter at the objective entrance pupil remains constant because it is a conjugate plane of the DM.	3.3.1
Does FOV size depend on the DM curvature?	Yes, for our implementation, there is a 2% change in FOV size between the upper and lower limits in the z-stack separated by 57 μm.	3.3.2
Does the axial scanning range depend on the objective NA?	Yes, if we use an objective with lower NA, the scanning range increases, but at the expense of spatial resolution.	3.3.2
Is the image plane flat as DM curvature changes?	The image plane, where 2P fluorescence occurs, is not flat because of field curvature induced by DM curvature. The deeper we image, the smaller the radius of curvature of the image plane.	3.3.2
Does DM-based axial scanning change the WFE of the system?	Yes, in an implementation-dependent manner. Changing the incoming beam wavefront to the objective during DM-based axial scanning can balance or increase other aberrations.	3.3.3
*Experimental characterization*		3.4
How does the PSF change with DM curvature?	DM curvature impacts on the lateral PSF full width at half maximum (FWHM) are marginal, while the axial FWHM almost doubles as DM curvature changes.	3.4.1
Is the axial scanning linear with the defocus?	As the A-parameter increases, the image plane is linearly shifted along the z axis to deeper layers.	3.4.2
Does 2P fluorescence intensity change with DM curvature?	2P fluorescence intensity changes as a function of DM curvature. This dependence was calibrated and could be corrected by the Pockels cell.	3.4.3

### 3.3 Optical Model Analysis

Different analyses of the optical model were conducted to evaluate the optical design and performance as DM-based axial scanning was used.

#### 3.3.1 Vignetting

**Fig. 6:**
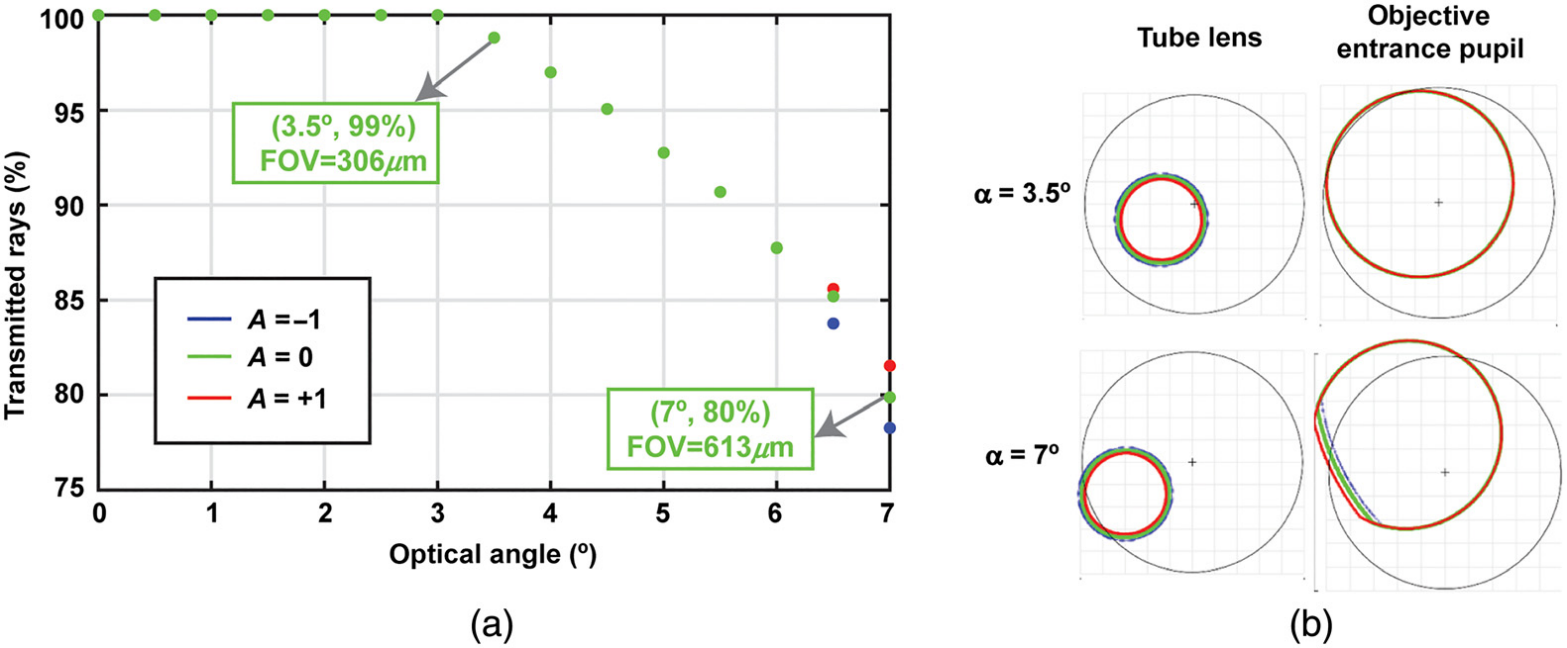
(a) Vignetting analysis: transmitted rays as a function of the optical angle of the GMs, for three DM curvatures. Two examples of FOV size are marked with gray arrows. (b) Beam footprint at the tube lens and the objective entrance pupil. Each row corresponds to a configuration with a different galvanometer optical angle (3.5 deg and 7 deg). Colors correspond to a specific DM curvature (given by the A-parameter). Color legend as (a). Black circle shows the aperture size at each plane (28 and 13.8 mm, respectively, for the tube lens and the objective entrance pupil).

We carried out a vignetting analysis using multiple configurations: first, the GMs were scanned at different angles in the x and y direction and second, the DM radius of curvature was adjusted. In this analysis, we studied the worst-case scenario in terms of vignetting, which is at the image corners. Figure 6(a) shows the number of transmitted rays reaching the sample as we increased the optical angle on both GMs for three DM curvatures (A=−1, 0 and +1). Figure 6(b) shows the beam footprint at two critical planes in which clipping occurs (tube lens and objective entrance pupil). This representation is shown for two optical angles in rows (3.5 deg and 7 deg) and for three DM curvatures using the same color legend of Fig. 6(a).

For the 3.5-deg case, vignetting occurs because the objective entrance pupil is clipping the beam due to the beam walk generated by the GMs [Fig. 6(b)]. This beam walk occurring at the objective entrance pupil is because this plane is a conjugate plane of the intermediate plane between both galvanometers, causing a small shift of the beam footprint at the pupil plane as a function of the scanning angle. In particular, at 3.5 deg, 99% of the rays reach the sample for all DM curvatures. From 3.5 deg to 6 deg, vignetting increases with optical angle and is independent of DM curvature. In these configurations, vignetting only occurs at the objective entrance pupil, in which the beam size is the same for all DM curvatures because that is a pupil plane [Fig. 6(b), second column]. From 6.5 deg, vignetting increases with the optical angle and is dependent on the DM curvature. In this region, the tube lens starts clipping the beam in addition to the objective entrance pupil [see second row in Fig. 6(b)]. Because the tube lens plane is not a pupil plane, the beam diameter is DM curvature-dependent, causing vignetting as a function of DM curvature. Specifically, the percentage of rays transmitted through the system at 7 deg decreases to 78%, 80%, and 82% for the DM configurations A=−1, 0, and +1, respectively.

#### 3.3.2 Optimization and axial scanning capability

Next, we modeled the 2P z-stack using DM-based axial scanning. We investigated the optical model with various DM curvatures and determined the image plane axial position ($z_{i}$) in which the spot size was minimum to find the location where the 2P absorption occurs. We noticed that the z position was different for on-axis or off-axis configurations. These preliminary results led us to carry out a two-variable optimization to minimize the image spot size root-mean-square (RMS). The variables used were the axial position and radius of curvature of the image plane. In addition, this optimization was conducted simultaneously for nine configurations along the FOV diagonal corresponding to different optical angles for the GMs, covering an angular range from −3.5  deg to +3.5  deg. Thus, in each optimization process, on-axis and off-axis configurations equally distributed along the FOV diagonal were considered. Finally, we repeated the spot size optimization process for different DM curvatures. Note that this optimization process was used to have a better characterization of the 2P imaging process, but not to physically modify the optical system.

Table 2 shows optimization results for three DM curvatures (A=−1, 0, and +1), corresponding to the upper, center, and lower layer of the z-stack. Using these, we determined that the z-stack depth, or axial scanning range (Δz), is 57  μm. There is an asymmetry in the axial scanning capability: the most superficial layer we can image is at −28  μm, whereas the deepest layer is at +29  μm. The asymmetry is a result of utilizing part of the DM stroke for correcting the initial bow of the DM surface (Fig. 2), reducing the available stroke for axial scanning control. We next observed that the image plane is curved, and its radius of curvature decreases as we imaged at deeper layers (A>0). This is the result of field curvature, a known aberration in optical microscopy for non-paraxial systems,^35^ which has been reported in publications analyzing its impact in 2P microscopy.^39^^,^^40^

**Table 2:** Optimization parameters (depth and radius of curvature of the image plane) when running a minimization of the chief ray RMS PSF radial size for nine configurations along one of the diagonals in the FOV. Field curvature in the last row.

DM	A-parameter	−1	0	+1
	Radius of curvature: $R_{\mathrm{DM}}$ (mm)	−746	∞	+746
Image plane	Axial position: $z_{i}$ (μm)	−28	0	+29
	Radius of curvature: $R_{i}$ (mm)	65.68	15.84	7.26
Field curvature (μm)		0.34	1.34	3.02

A larger axial scanning range could theoretically be achieved using a microscope objective with a smaller NA. As an exploratory analysis, we replaced the 1.0 NA objective in the model with one of 0.8 NA, which led to an axial scanning range of 111  μm. However, the spatial resolution is reduced, resulting in a trade-off between those variables.

Finally, we evaluated the FOV size dependency with the DM curvature and found a change of FOV size of 2% between the upper and lower layers of the 57-μm deep z-stack (as we image at deeper layers, A>0, FOV size increases).

#### 3.3.3 Wavefront error

**Fig. 7:**
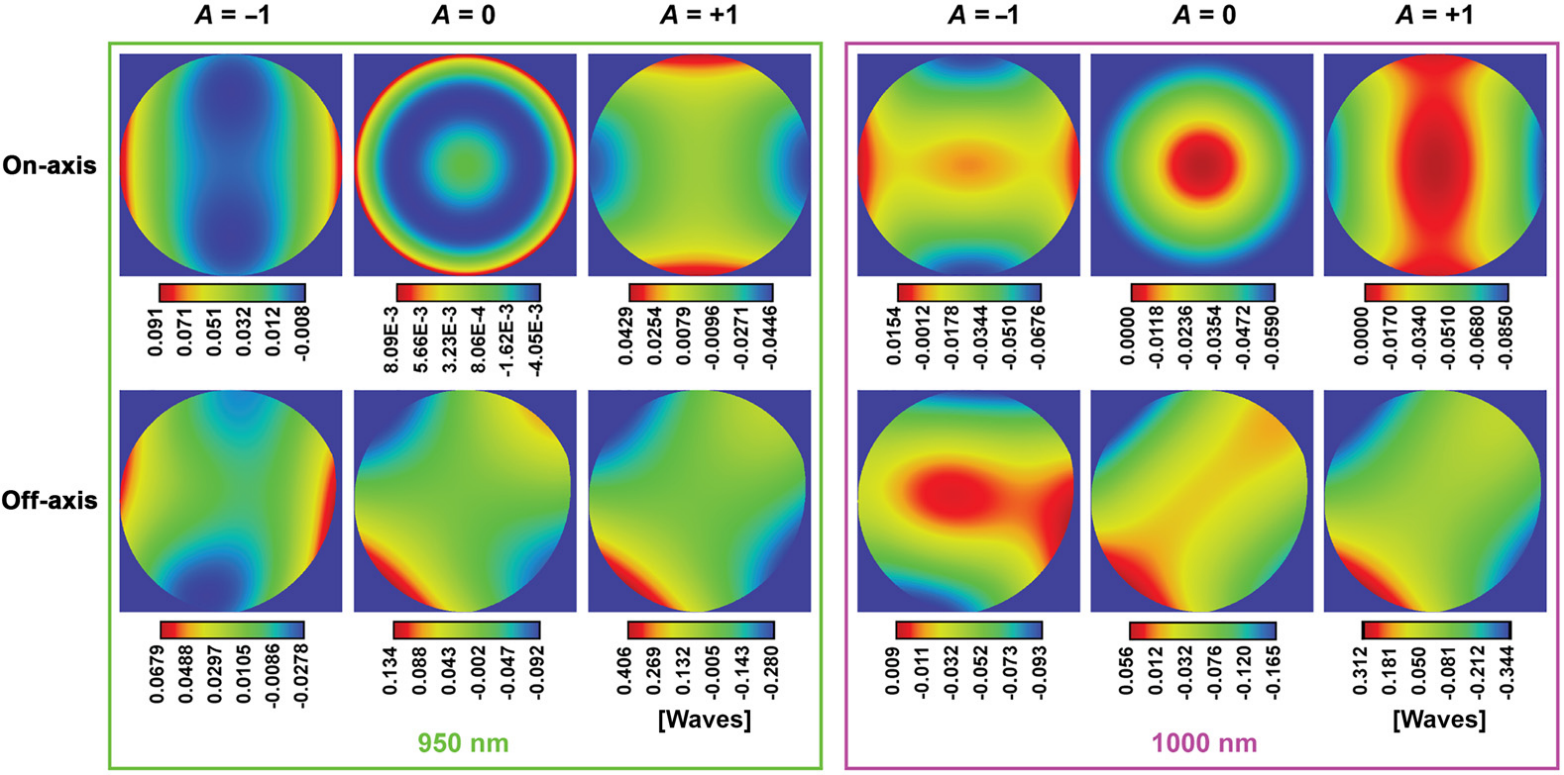
WFE map across the pupil for six different configurations and two illumination wavelengths (950 and 1000 nm). WFE map units in waves.

Next, we analyzed the WFE contribution of the scanning module shown in Fig. 7 as a function of pupil coordinates. This analysis was conducted using six different configurations and two illumination wavelengths (950 and 1000 nm). Each row in Fig. 7 corresponds to an optical angle generated by the GM (α): the first row shows on-axis configurations (α=0), corresponding to the center of the FOV and the second row shows off-axis configurations (α=3.5  deg), representing one of the four corners of a 306-μm FOV. Each column corresponds to a DM curvature: maximum negative curvature (A=−1), flat (A=0) and, maximum positive curvature (A=+1).

We observed a symmetry of the WFE map with respect to the x and y pupil coordinates for the on-axis configurations. We determined that the WFE PV, shown in Table 3, is smaller than a quarter of a wave (diffraction-limited) in most configurations, except for the off-axis configuration with maximum positive DM curvature, when the WFE PV reaches 0.7 waves. This was true for both wavelengths analyzed.

**Table 3:** WFE PV (Fig. 7); units in waves.

Wavelength	950 nm			1000 nm		
A-parameter	−1	0	+1	−1	0	+1
On-axis	0.098	0.012	0.088	0.083	0.059	0.085
Off-axis	0.096	0.226	0.686	0.103	0.221	0.656

Our optical model considers the microscope objective as a paraxial lens because the actual optical model or a black box of the system was not released by the vendor. As a result, the real WFE maps will be larger than those plotted in Fig. 7 due to residual objective aberrations and non-collimated illumination to the infinity-corrected objective resulting from DM curvature changes. However, in Sec. 3.4, we present the experimental characterization of the implemented microscope, in which the contributions of all the elements of the optical system are considered, including the microscope objective.

### 3.4 Experimental Characterization

In this section, we perform an experimental characterization of the 2P microscope with the aim of evaluating the optical performance of the instrument as we scan along the z axis with the DM.

#### 3.4.1 Point spread function

The experimental PSF was calibrated for different DM curvatures. To do so, we imaged 0.1-μm-diameter fluorescent microspheres (F8803, Invitrogen). A solution of 4% low melting point agarose and water was used to dissolve 0.2% of the microspheres. This solution was solidified on a glass side and then coverslipped. These microspheres have a nominal one-photon excitation and emission wavelengths of 505 and 515 nm, respectively. Thus, to ensure a 2P absorption, the microsphere sample was illuminated with the pulsed laser at 1010 nm. A long-pass filter at 660 nm was used before the microscope objective, transmitting the NIR laser beam light to the sample, and reflecting the visible emitted light from the sample to the detector.

The protocol used to characterize the PSF was the following: The sample was aligned so nanobeads were imaged at the center of the FOV. Then, a z-stack composed of 81 xy images was acquired. Between frames, the microscope objective was moved in steps of 0.3  μm along the z axis using a linear motorized stage (DRV014, Thorlabs), while the DM was kept at the same curvature. The FOV size was 10.8  μm with a pixel size of 0.04  μm and the frame rate during acquisition was 4.25 Hz.

**Fig. 8:**
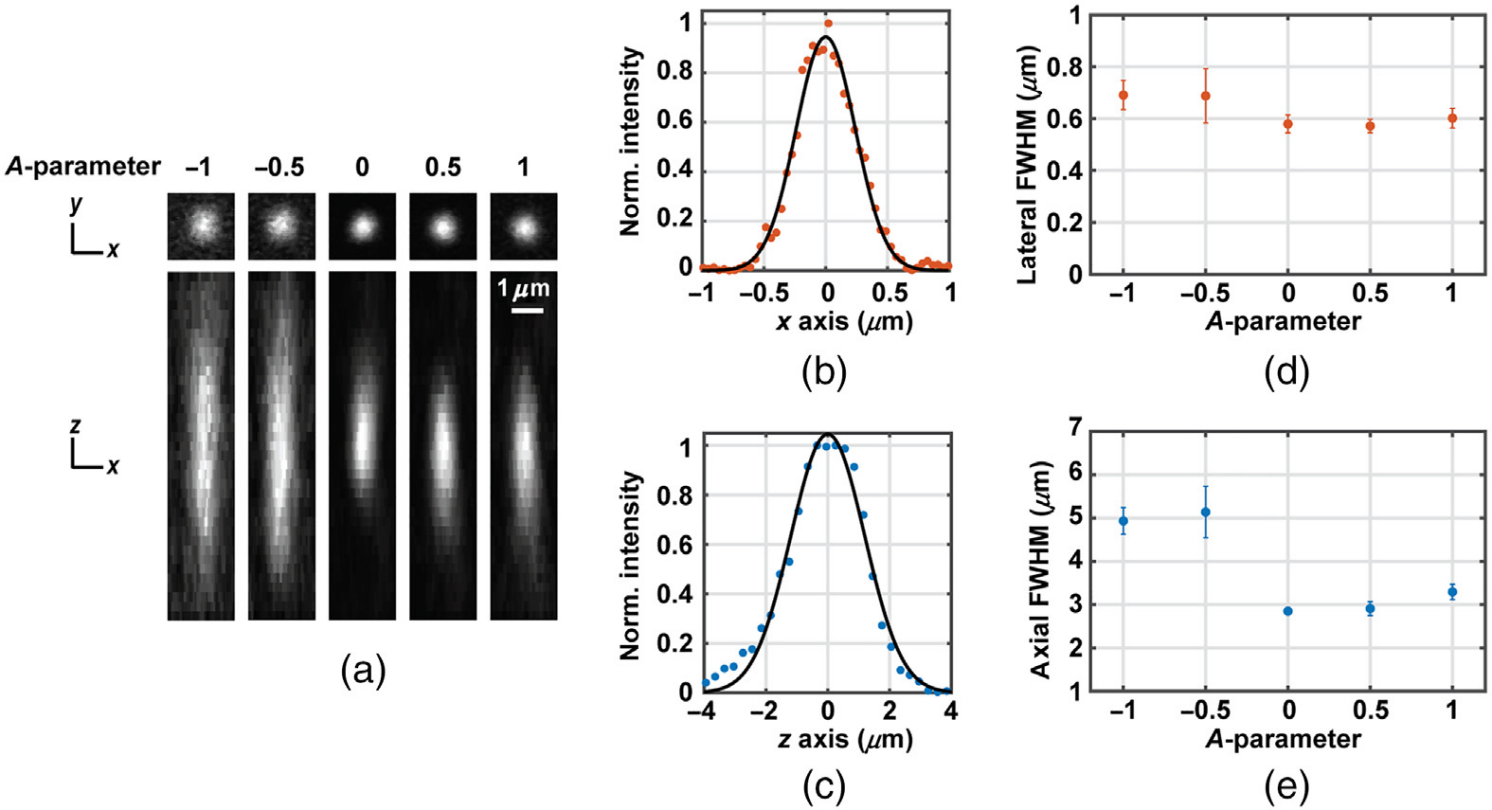
PSF experimentally determined using 0.1-μm-diameter microspheres. (a) Images at the center of the FOV on plane xy (first row) and on plane xz (second row), for five DM curvatures (A=−1, −0.5, 0, 0.5, and 1). (b) and (c) Example of lateral and axial normalized intensity cross-sections for the DM at flat configuration (A=0). Points correspond to experimental data and black line to the Gaussian fit used to calculate FWHM. (d) and (e) Lateral and axial FWHM as function of the DM curvature (given by the A-parameter). Mean and standard deviation of six different beads. Lateral FWHM considers x and y cross-sections Gaussian fits.

Figure 8(a) shows the experimental PSF cross-sections images corresponding to planes xy and xz for different curvatures of the DM, with the A-parameter ranging from −1 to +1 in steps of 0.5. Experimental data were processed using MetroloJ,^41^^,^^42^ an ImageJ plugin to characterize the instrument’s PSF. The algorithm fits each cross-section profile to a Gaussian function [see an example for A=0 Figs. 8(b) and 8(c)] and determines the resolution in terms of full width at half maximum (FWHM) for each axis. To calculate the lateral resolution, we averaged both x and y FWHM. Axial and lateral resolutions were numerically characterized for all five analyzed DM curvatures. Six different beads were measured for each DM curvature, and the mean and standard deviation of the lateral and axial FWHMs were calculated and represented in Figs. 8(d) and 8(e), respectively. Experimental data show that axial resolution is more sensitive to a change in the DM curvature. In particular, axial FWHM increases up to 80% and 73%, respectively, at A=−0.5 and −1 compared to the minimum FWHM (at A=0 and 0.5). On the other hand, lateral FWHM remains within 21% in A=−0.5 and −1 compared to the minimum FWHM (at A=0 and 0.5). Furthermore, the changes in the PSF dimensions are not symmetric for positive and negatives values of A. While the axial FWHM rapidly increases by a factor of 1.7 for A=−0.5, the PSF FWHM remains almost unchanged for A=+0.5. Our hypothesis is that non-collimated illumination of an infinity-corrected objective induces additional aberrations to the transmitted wavefront besides the pure defocus term desired to achieve axial scanning. The asymmetric behavior of the PSF could be the result of the DM curvature balancing residual objective aberrations for A>0, but increasing those aberrations for A<0. While we were not able to test this hypothesis in the optical model, we have identified the fraction of the axial scanning range in which the PSF performance is preserved (0<A<0.5), so the user can trade axial range for PSF quality if the entire range is needed.

#### 3.4.2 Experimental axial scanning capability

**Fig. 9:**
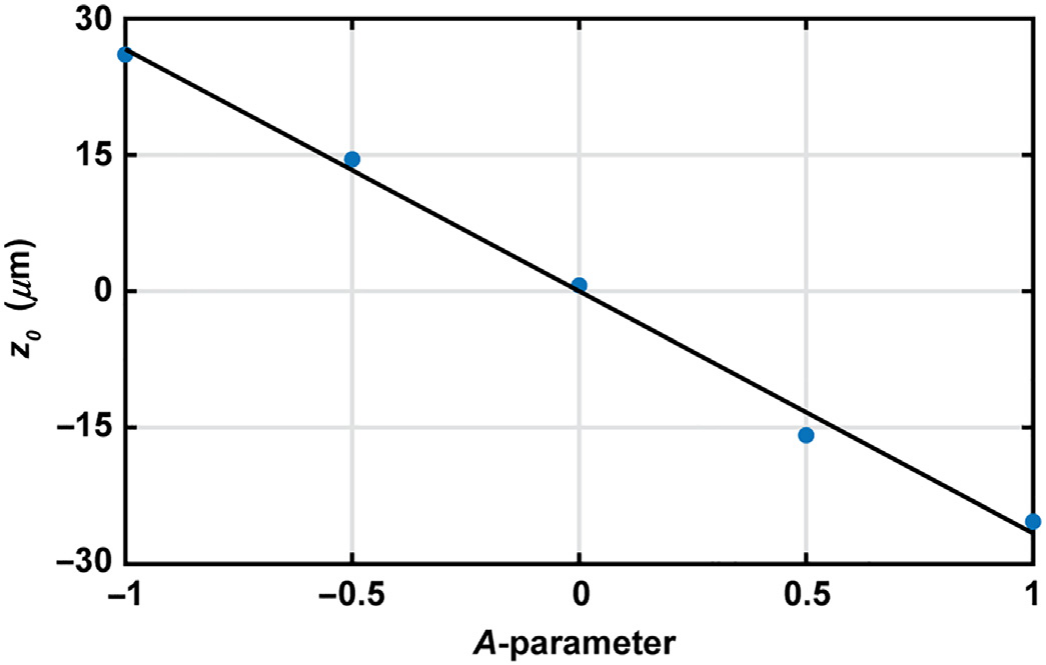
PSF z-profile peak position ($z_{o}$) as a function of the A-parameter in points. Linear fit in continuous line corresponding to Eq. (5), with a $R^{2}=0.995$.

To measure the microscope axial scanning range, we determined the PSF z-profile peak position ($z_{o}$) as a function of the A-parameter for the five experimental PSFs measured in Sec. 3.4.1. Experimental data are plotted in points in Fig. 9, and a linear regression fit is added. This linear fit, describing how the DM axially scans in depth along the sample as a function of the DM curvature, has the following expression:

z=−26.4A. (5)

We determined that the total experimental scanning range of our system (Δz) is 52.8  μm, obtained from the z difference between A equal −1 and +1 using Eq. (5). This experimental scanning range is consistent with the range predicted by the optical model (57  μm) in Sec. 3.3.2. The difference between the predicted and observed scanning range of 7.4% (4.2  μm) may be explained by a combination of factors in which the real system differs from the modeled one, such as DM inter-actuator coupling resulting in more prominent DM shapes (when A is far from 0), or the effect of non-collimated illumination on the microscope objective.

#### 3.4.3 Intensity as a function of A-parameter

**Fig. 10:**
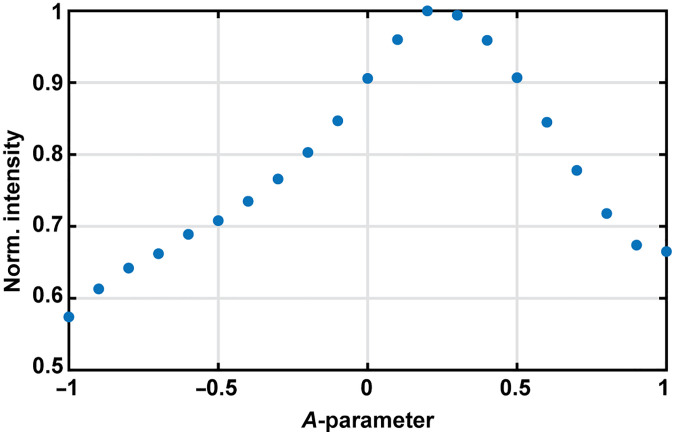
Normalized average intensity over the FOV as a function of the DM curvature (given by the A-parameter) when imaging a uniform fluorescence slide.

To understand the 2P intensity dependency on the DM curvature, we performed the following experiment. A uniform fluorescence slide was imaged with the 2P microscope at different depths using DM axial scanning by changing the A-parameter in steps of 0.1 and with an FOV of 270  μm. Figure 10 shows the normalized mean intensity along the z-stack as the DM curvature changes. We checked that the fluorescence intensity at different depths in the sample was constant by moving the objective with a linear stage; this test confirmed that intensity variations observed in Fig. 10 are due to DM curvature changes. We determined that the maximum intensity does not occur with the flat DM configuration, but rather for A=0.2. This supports our hypothesis in Sec. 3.4.1 which assumes that the wavefront change introduced by the DM at A=0.2 partially balances aberrations present in the system by optimizing the 2P fluorescence signal. This value is consistent with the optimal A regime (0 to 0.5) that maximizes the axial and lateral resolutions as described in Sec. 3.4.1.

The maximum FOV intensity is found at A=0.2, and the lowest intensity value at A=−1, consistent with a gradual axial PSF elongation observed empirically as the A-parameter is decreased to negative values. Once the intensity variations as a function of the DM curvature (Fig. 10) are calibrated, one could tune the input light beam power using the Pockels cell to normalize the final fluorescence intensity. Alternatively, an adaptive optics algorithm could be implemented using the DM in which wavefront correction terms could be added to defocus for axial scanning control. The resulting PSF would be sharper, and thus achieve a more uniform fluorescence intensity as DM curvature changes. This approach has been proven,^29^ but is beyond the scope of the current work.

## 4 Experimental Measurements

The instrument has been tested with three different samples: pollen grains, a fixed mouse brain slice and, lastly, *in vivo* mouse cerebral cortex. All procedures were carried out using mice, in accordance with protocols approved by the University of California, San Francisco Institutional Animal Care and Use Committee.

### 4.1 Pollen

**Fig. 11:**
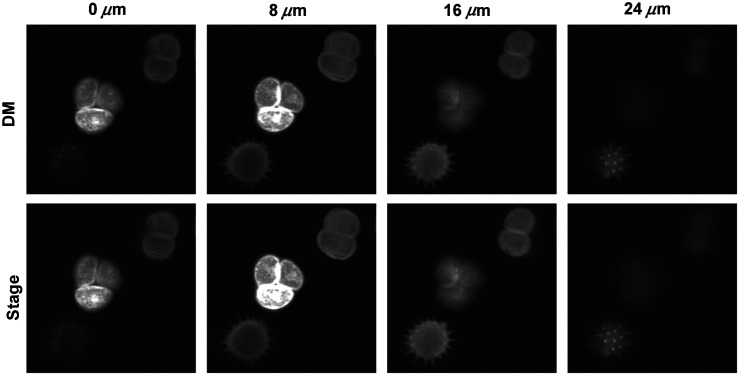
Pollen grains imaged at 1040 nm. 2P images acquired at four depths, using the DM (first row), and the objective linear stage (second row). FOV=104  μm×104  μm.

The sample was composed of grains of different types of pollen. A z-stack of four frames at different depth was acquired, each plane was 8  μm apart. The change in depth during the acquisition was performed first using the DM system and, second, moving the microscope objective with a motorized linear stage in the z-axis while keeping the DM in its flat configuration. Both z-stacks, shown in Fig. 11, were acquired at a frame rate of 1.1 Hz while illuminating the sample at 1040 nm and collecting all emitted light below 660 nm. Experimental images from Fig. 11 show that the DM system used for axially scanning in 2P microscopy provides excellent and comparable results to the ones acquired with the traditional linear stage which mechanically moves the objective.

### 4.2 Fixed Brain Slice

Next, we imaged a fixed brain slice from an Aldh1l1-tdTomato mouse in which the fluorescent protein tdTomato is expressed solely in astrocytes. The sample was prepared through intracardial perfusion with 4% paraformaldehyde, which was then sliced coronally at 300  μm. To amplify the fluorescent signal, the tissue samples were then stained via immunohistochemistry and mounted on a glass slide.

**Fig. 12:**
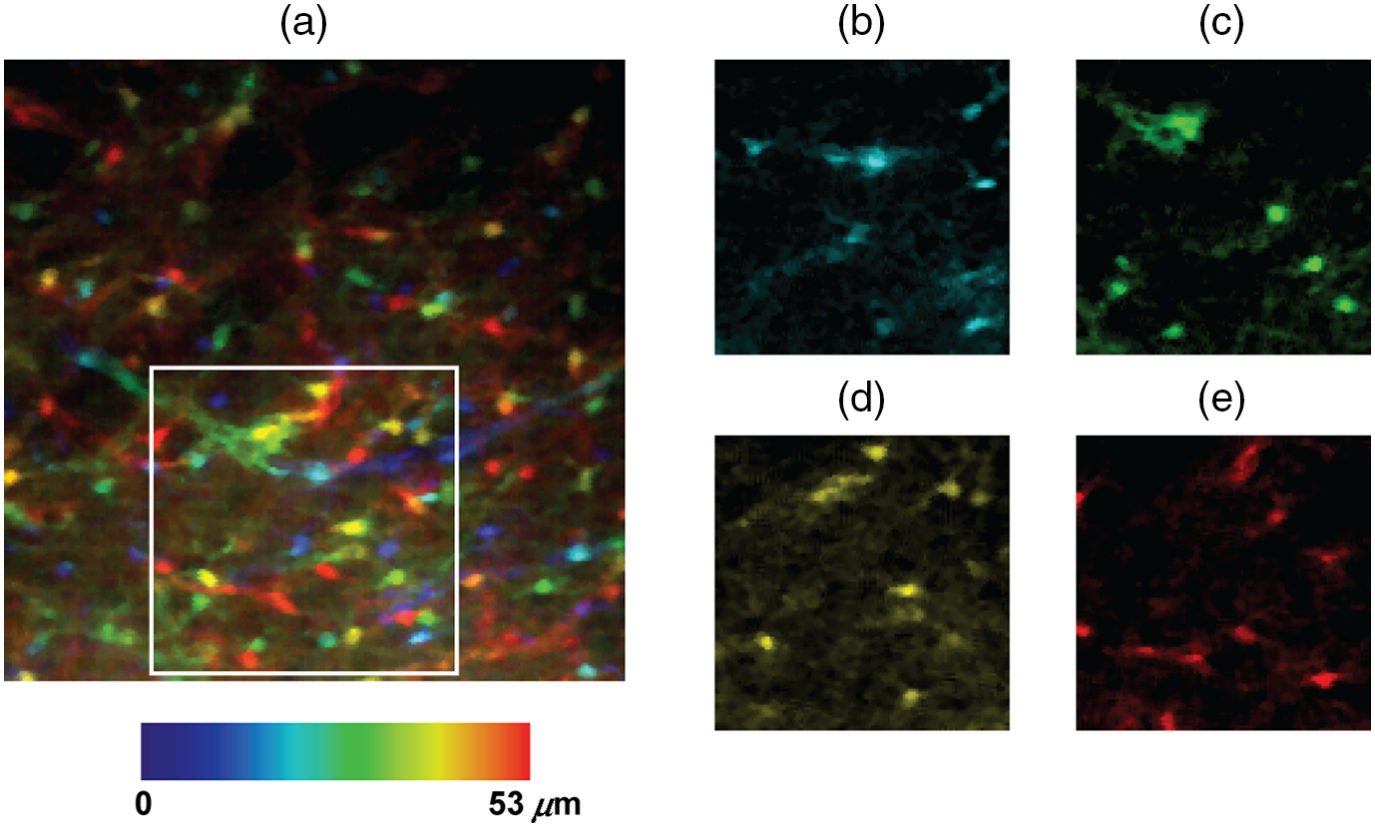
Fixed brain slice (expressing tdTomato in astrocytes) illuminated at 1040 nm. 2P z-stack acquired using the DM to change the depth in steps of δz=0.3  μm. (a) Maximum intensity z-projection of thez-stack, with color-coded depth. FOV=288  μm×288  μm×53  μm. White box is represented for four depths: (b) 22, (c) 32, (d) 42, and, (e) 52  μm.

Brian slices were illuminated at 1040 nm, and images were recorded at a frame rate of 1.1 Hz. A z-stack of 201 frames was acquired using the DM, in steps of 0.3  μm. Figure 12(a) shows a maximum intensity z-projection of the recorded z-stack, with color-coded depth. There is a complex distribution of astrocyte somas across the whole FOV (288  μm×288  μm×53  μm). We show a detailed view of the white box in Fig. 12(a) at four given depths, 22, 32, 42, and 52  μm, respectively, in Figs. 12(b), 12(c), 12(d), and 12(e).

### 4.3 *In Vivo* Brain Imaging

Finally, we used the novel 2P microscope for *in vivo* brain imaging. We imaged EAAT2-tdTomato mice to visualize both the soma and the fine processes of astrocytes. On the day of imaging, mice were anesthetized with urethane (100  mg/ml) and a craniotomy (~3 mm) was made over the imaging area. A 4% agarose solution was placed upon the craniotomy and a glass window was secured into place using dental cement. During the imaging session, mice were kept anesthetized and head-fixed under the microscope objective. We illuminated at 1020 nm, and the image acquisition was taken at 1.1 Hz. A z-stack of 201 frames was obtained, with steps of 0.3  μm. The region of interest (ROI) was in layer 2/3 of the primary visual cortex.

**Fig. 13:**
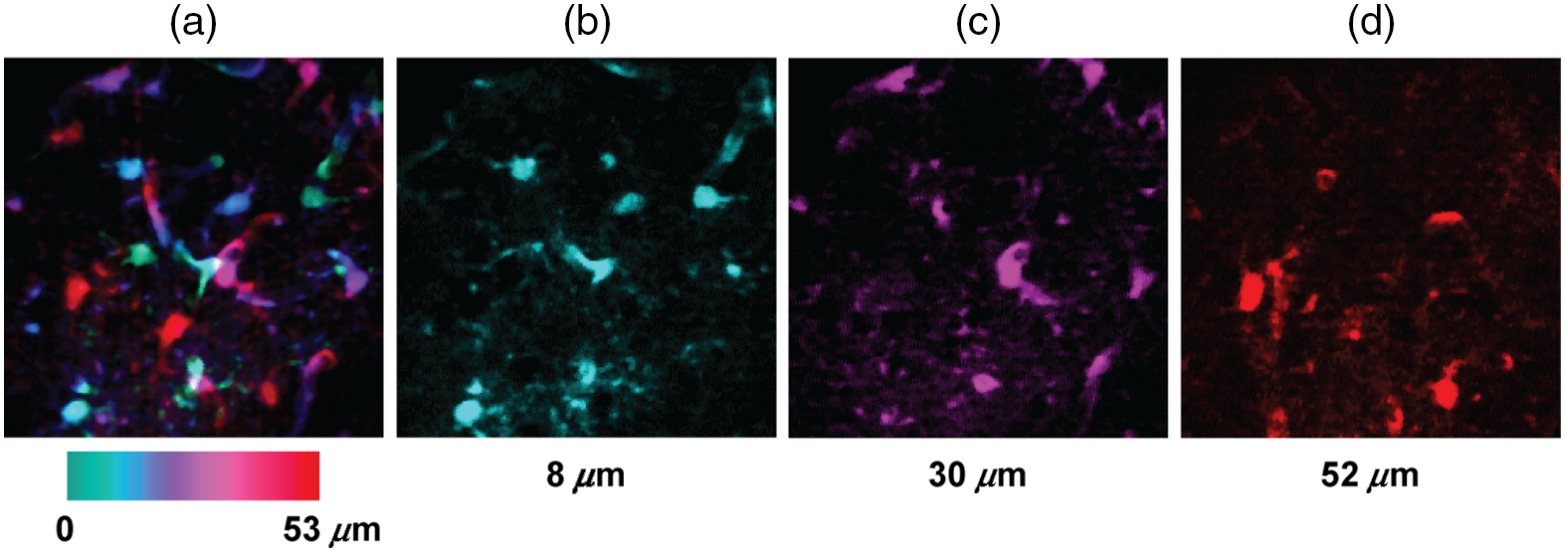
*In vivo* brain imaging of astrocytes illuminated at 1020 nm. 2P z-stack acquired using the DM to change the depth in steps of δz=0.3  μm. (a) Maximum intensity z-projection color-coded by depth. FOV=306  μm×306  μm×53  μm. Single frames taken at depths of (b) 8, (c) 30, and, (d) 52  μm.

Figure 13(a) shows the maximum intensity z-projection of the recorded z-stack, in which we color-coded depth. As z increases, the microscope images astrocytes at deeper layers. The FOV is 306  μm×306  μm×53  μm. In addition, Figs. 13(b), 13(c), and 13(d) show the same ROI but at a single specific depth: 8, 30, and 52  μm, respectively.

## 5 Conclusions

This work presents the optical design and implementation of a 2P microscope which incorporates a DM for axial scanning. During the design and characterization process, we studied the behavior of the system as a function of the optical parameters selected. Our implementation was capable of acquiring z-stacks of 53  μm depth, with a fine step size (0.3  μm). We experimentally characterized the PSF size as a function of the DM curvature. Lateral spatial resolution remains within 21% of the minimum FWHM, while axial spatial resolution can increase up to 80% as the DM curvature changes. We calibrated the 2P fluorescence intensity function with the DM curvature change and, in the future, this could be corrected using the Pockels cell. Our findings are reported in Table 1, to be used by other groups developing similar instruments. For example, we found (1) that vignetting caused by DM-based axial scanning occurs as the FOV increases due to beam walk on the tube lens, (2) the objective filling condition remains constant while changing the DM curvature, (3) the FOV size changes 2% between the upper and lower layers of the z-sack, (4) the 2P image plane is not flat and its radius of curvature depends on the DM curvature, and (5) DM-based axial scanning changes the WFE of the system.

The instrument was then experimentally tested by measuring three biological samples. First, pollen grain imaging demonstrated that DM-based axial scanning produces equivalent images as those obtained by mechanically moving the microscope objective along the z axis. Two neurobiological samples with fluorescent astrocytes were imaged: fixed mouse brain slices and *in vivo* cerebral cortex of anesthetized mice. Both imaging experiments allowed us to visualize and map the morphology of fluorescent astrocytes tangled in depth, showing the capability of this DM-based technology for axial scanning in brain imaging.

The system we present here is especially advantageous with respect to other volumetric or axial scanning (xz axis) 2P microscopes when imaging more than a few z-planes with a time resolution compatible to neurobiological processes. The DM used in our system has a fast response time (<100  μs), allowing for axially scanning with a small axial step size and fast acquisition frame rate. For example, when combined with a GM scanning the x axis at 1.5 KHz, the microscope would scan an xz plane with a resolution of 512×512  pixels with a dwell time of 1.2  μs, for an acquisition frame rate of 2.5 Hz. Speed could be increased using a resonant mirror. For example, assuming a resonance frequency of 8 KHz, the microscope could reach frame rates of 7 Hz for the xz plane. Future work will focus on the precise synchronization between the DM-based axial scanning and the 2D scanning by the GMs set. This will open up the axial dimension for fast 2P imaging at multiple depths, which has the potential to resolve temporally and spatially complex and heterogeneous dynamics, including those observed when imaging astrocytic ${\mathrm{Ca}}^{2+}$ sensors, extracellular neurotransmitter sensors, and membrane-targeted voltage sensors in neurons. In addition, future work will include the use of adaptive optics with the DM—simultaneously with axial scanning control—to correct aberrations and mitigate the effect of non-collimated illumination to an infinity-corrected objective.
